# Distribution patterns and drivers of nonendemic and endemic glires species in China

**DOI:** 10.1002/ece3.9798

**Published:** 2023-02-07

**Authors:** Lei Meng, Lizhi Zhou

**Affiliations:** ^1^ School of Resources and Environmental Engineering Anhui University Hefei China; ^2^ Anhui Biodiversity Information Center Anhui University Hefei China

**Keywords:** endemic species, environmental variables, glires, multivariate analysis, richness patterns

## Abstract

Spatial patterns and determinants of species richness in complex geographical regions are important subjects of current biogeography and biodiversity conservation research. Glires are small herbivorous mammal species with limited migratory ability that may serve as an indicator of biodiversity and ecosystems. Herein, we aimed to evaluate how multiple ecological hypotheses could explain the species richness patterns of glires in China. Initially, we constructed a mapping grid cell operating units of 80 × 80 km^2^ which covered China's land mass and mapped the distribution ranges of the 237 glires species that had recorded. The glires taxa were separated into three response variables based on their distribution: (a) all species, (b) nonendemic species, and (c) endemic species. The species richness patterns of the response variables were evaluated using four predictor sets: (a) hydrothermal characteristics, (b) climatic seasonality, (c) habitat heterogeneity, and (d) human factors. We performed regression tree analysis, multiple linear regression analysis, and variation partitioning analyses to determine the effects of predictors on spatial species patterns. The results showed that the distribution pattern of species richness was the highest in the Hengduan Mountains and surrounding areas in southwest China. However, only a few endemic species adapted to high‐latitude environments. It was found that there are differences about the determinants between nonendemic and endemic species. Habitat heterogeneity was the most influential determinant for the distribution patterns of nonendemic species richness. Climatic seasonality was the best predictor to determine the richness distribution pattern of endemic species, whereas this was least affected by human factors. Furthermore, it should be noted that hydrothermal characteristics were not strong predictors of richness patterns for all or nonendemic species, which may be due to the fact that there are also more species in some areas with less precipitation or energy. Therefore, glires are likely to persist in areas with characteristics of high habitat heterogeneity and stable climate.

## Graphical abstract

This study explores the distribution pattern of glires and their endemic and nonendemic species. We evaluated how multiple ecological hypotheses could explain the spatial richness patterns of glires in China.

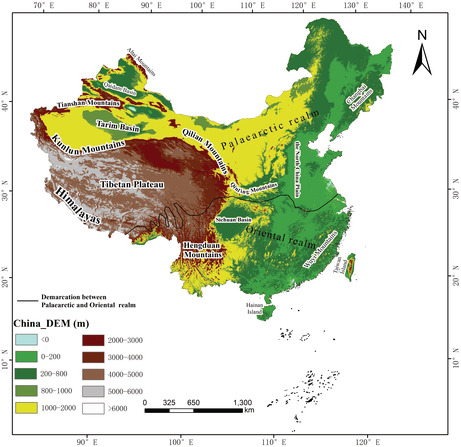

## 1 INTRODUCTION

An understanding of large‐scale spatial distribution patterns of species richness and their determinants is essential for biogeography and biodiversity conservation. Species richness is also a fundamental measure of community and regional diversity, and the basis for the construction of many ecological models and conservation strategies (D'Antraccoli et al., 2019; Gotelli & Colwell, 2001; Jenkins et al., 2013). How interactions of the modern environment, evolutionary history, and ecological processes shape the patterns of species richness distribution remains an interesting but controversial topic in biogeography. In recent decades, species distribution has been greatly affected by climate change and human activities (Li et al., 2015; Mi et al., 2022). Thus, clarifying species richness distribution patterns is important for conserving biodiversity and providing basic information for management or societal decisions (Holt et al., 2018). Species are not randomly distributed over the land surface; rather, their distribution patterns are based on climate, topography, biotic forces, and anthropogenic influences in recent decades (Li et al., 2015; Xu et al., 2019). Consequently, various theories and hypotheses have been developed to explain how geographical patterns of species richness are formed.

China harbors 271 glires species (Wei et al., 2021), reflecting a global hot spot of glires species richness. The mammalian Class of Glires, contains the Orders Rodentia and Lagomorpha, which have limited migratory ability, small size and are sensitive to the environment, thereby representing a useful indicator of biodiversity. Most species are phytophagous, opportunistically feeding and foraging on diverse vegetation types which provides them with the capacity to adapt to anthropogenic habitats. China also has rich environmental gradients, ranging from tropical to boreal zones, forests to deserts, and high mountains to depressions below sea level (Xu et al., 2019). The rich diversity of glires in China may be favored due to the climatic and geographical variations of its vast ecological region (Hu et al., 2017; Wu et al., 2013). In any large ecological region, species richness distribution may be driven by two or more environmental gradients (Terribile et al., 2009). In addition, hydrothermal characteristics, climate seasonality, habitat heterogeneity, and human factors are directly related to animal diversity and are the determinants of regional species richness changes (Amori et al., 2011; Lewin et al., 2016; Mi et al., 2022). Therefore, in this study, we sought to evaluate the relative role of these factors in explaining patterns of glires species richness in China.

The hydrothermal characteristics hypothesis is most commonly discussed for explaining species richness patterns (Hawkins et al., 2003; Pandey et al., 2020). This hypothesis states that the availability of energy and precipitation determine the total plant resources that influence biological activity and that total plant resources subsequently determine changes in biodiversity (Jimenez‐Alfaro et al., 2016). Second, habitat heterogeneity hypothesis is a synergistic relationship between species distribution and topographic variation. The existence of environmental or resource heterogeneity may produce high ecological niche diversity, allowing species to coexist over large spatial scales. As plant diversity increases, species richness increases and is highly scale‐dependent within a landscape, and species richness gradients occur with local and regional species replacement (Stein et al., 2014, 2015). Third, seasonal changes in climate and unsystematic changes in daily maximum and minimum temperatures may increase organisms' thermal tolerance levels, enabling them to become geographically widespread (Mi et al., 2022). Finally, human‐induced environmental changes, such as habitat fragmentation, land‐use changes, and disturbances, can lead to habitat loss for species (Li et al., 2015; Xu et al., 2019). These hypotheses were based on different environmental factors, which explored the distribution patterns of species richness.

To determine the spatial distribution of species richness, previous studies have tested limited hypotheses (Barreto et al., 2019; Sun et al., 2020) and multiple hypotheses (Ding et al., 2019; Gebauer et al., 2018; Pandey et al., 2020). The interpretation of species richness patterns by a single variable or hypothesis is limited, as combined complex phenomena determine the distribution pattern of species richness. Thus, multiple modeling approaches are best suited for quantifying the contribution of various hypotheses to spatial richness distribution patterns. Moreover, there are studies that explained the distribution of glires in China (Xing, 2008; Zhou, 2000), and the mechanism that determines richness patterns were initially discussed. Chi et al. (2020, 2021) studied the distribution pattern of terrestrial mammal abundance in China and its relationship with environmental factors. Inevitably, these studies did not take sufficient account of glires distribution patterns, especially the endemic and nonendemic glires groups in China. Endemic species are those found only in specific locations or regions. They are usually restricted to a limited geographic range, with small ranges and population sizes, and sometimes with low genetic diversity and specific habitat requirements (Isik, 2011; Myers et al., 2000). Multiscale drivers and geographic distribution patterns of endemic species are also important topics in conservation biogeography due to their vulnerability to climate change and habitat degradation (Wu et al., 2016). In comparison, nonendemic species show a strong diffusion trend and a larger geographical range. It has been shown that there is a lack of consistency between all species or nonendemic species richness and endemic species richness (Lamoreux et al., 2006; Orme et al., 2005). Areas with high species richness may have many endemic species but not necessarily consistent patterns (Vetaas & Grytnes, 2002).

The prediction of species ranges can usually be achieved by several steps: collection of species distribution sites, based on species habitat use and habitat characteristics, expert mapping of species distributions, or inferring ranges from species distribution models (Guisan & Thuiller, 2005). At present, species distribution models (SDM) are frequently used in studies, because of their relative flexibility and good discriminative and predictive ability. Species distribution models can use the relationship between species distribution points and local environmental variables to predict the potential distribution areas of species (Abdulwahab et al., 2022; Sanczuk et al., 2022). China is a vast territory; hence, covering this geography with biological field surveys is not realistically possible. Therefore, SDM can guide future field surveys to a certain extent, provide references for further exploration and guide the discovery of potential distribution areas for species (Nguyen & Leung, 2022). Among the simulation methods of various distribution models, the maximum entropy model uses environmental variables and species distribution sites to calculate constraints in the case of a small sample size. It explores the possible distribution of maximum entropy under this constraint to predict the habitat suitability of species in the study area and may result in better simulation results than other models (Wang et al., 2021).

In this study, multisource glires data were used to analyze geographical distribution pattern and shed light on the maintenance mechanism of species richness in China. We divided glires into endemic and nonendemic species and assumed that the factors affecting endemic and nonendemic species distribution are different. We investigated the relative importance of hydrothermal characteristics, climatic seasonality, habitat heterogeneity, and human factors that may contribute to the distribution patterns of glires in China.

## 2 MATERIALS AND METHODS

### 2.1 Study area

**FIGURE 1:**
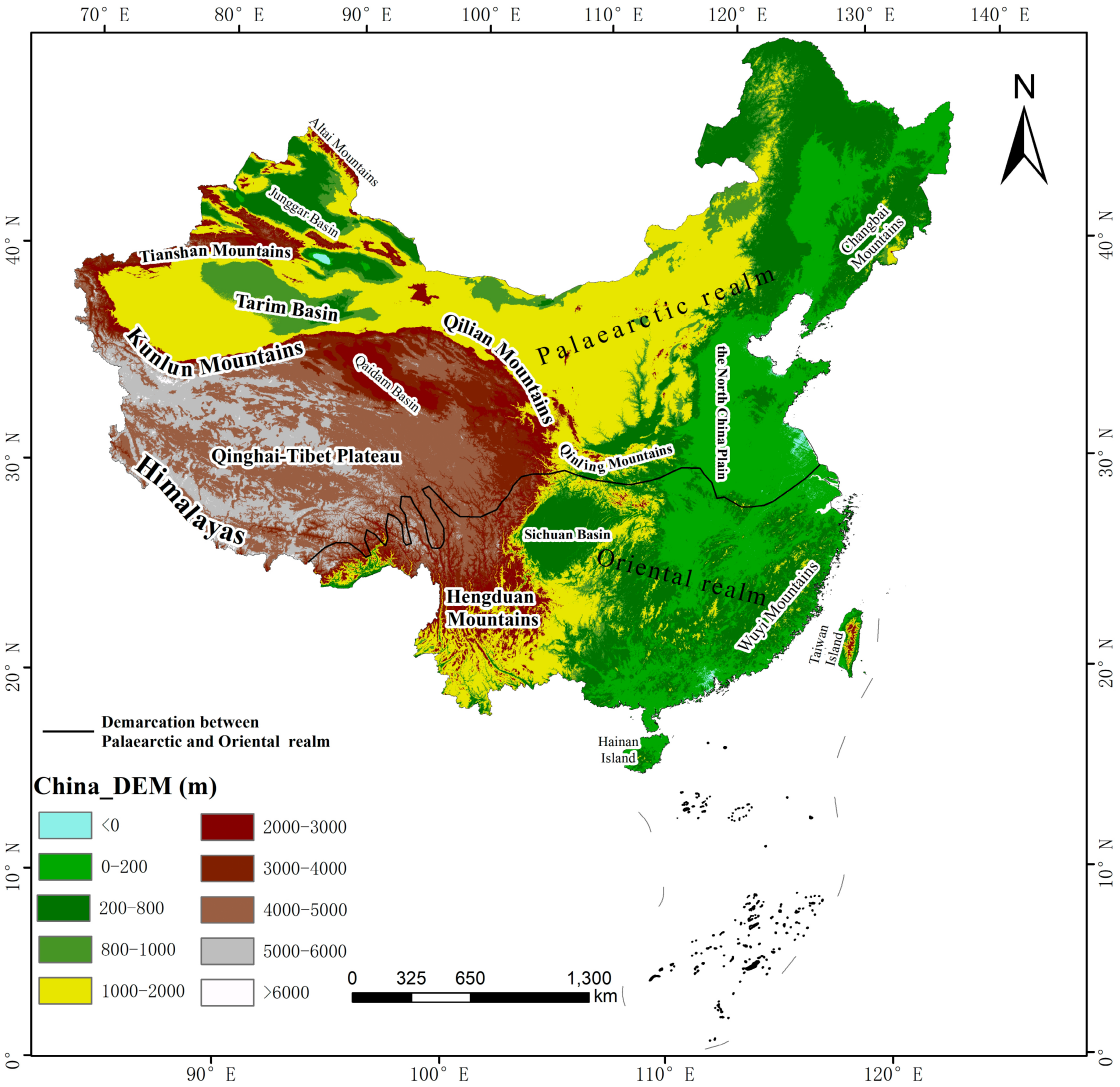
Topography and major regions of China.

The study encompassed all of China's land mass (Figure 1). Chinese mammalian fauna belongs to the Palearctic and Oriental realms, which can be further subdivided into seven biogeographic subregions (i.e., northeast China, north China, Inner Mongolia‐Xinjiang region, Qinghai‐Tibet region, southwest China, central China, and south China). In these ecogeographic zones, climate varies widely from the tropics to the cool‐temperate zone, with a clear division between dry and wet regions. Vegetation is also diverse, covering a wide range of zones, including rainforest, steppes, and desert. Referring to the previous studies (Xing, 2008; Zhou, 2000), we divided the Chinese territory into grid cell operating units of 80 × 80 km^2^ to eliminate the influence of area on species distribution patterns. Incomplete grid cells (<75% of the complete cell) present in the study area in the coastline and boundary areas were removed to prevent them from affecting the subsequent statistical analysis. A total of 1672 grid cell operating units were obtained.

### 2.2 Distribution pattern calculation

We compiled a database of glires species distribution based on the latest mammal list (Wei et al., 2021). Species distribution data were obtained from the following sources: (1) the research results of Zhou (2000) and Xing (2008); (2) National Zoological Museum of China, NZMC; (3) Global Biodiversity Information Facility (GBIF); and (4) distribution and collection records available in books or literature (Cheng et al., 2021; Ge et al., 2018; Jackson et al., 2022; Jiang et al., 2015; Li et al., 2019; Liu et al., 2019, 2020). All Chinese lands were used as study areas in this study, and species distribution data from outside China were excluded to ensure the successful operation of the model (Wang et al., 2021). In the end, we collected 21,089 valid distribution points for 237 species. There were 67 endemic and 170 nonendemic species (Jiang et al., 2015; Wei et al., 2021).

MaxEnt (v3.4.1) is used to predict the potential habitat of glires in China. The MaxEnt model requires at least five different coordinate values for each species to produce more accurate results (Mi et al., 2022); therefore, six points were used as the minimum criteria for calculating species distribution in this study. The potential habitats of 210 glires species with six or more distribution points were simulated using SDM to determine the potential species richness of glires in China. Based on the characteristics of distribution data and glires habits, 26 environmental variables were selected and converted to 1 km^2^ resolution. The five categories of predictors were climate, topography, vegetation, soil, and human activity intensity (Appendix S1). Chinese administrative vector boundaries were obtained from the Data Center for Resources and Environmental Sciences at the Chinese Academy of Sciences (RESDC) (http://www.resdc.cn).

The correlation of environmental variables was detected using the ENMTools (Warren et al., 2021) package in R 4.1.3 (http://www.r‐project.org). The variables that were not highly correlated (*r* < 0.7) were retained as variables of high biological significance for glires and subsequently used in the model prediction to reduce the complexity of the model (Appendix S2). The percentage of random test data was set to 25%, 10 submodels were generated using the bootstrap function of the MaxEnt model, and the average of the output of the 10 submodels on each image element was calculated as the final prediction result of the species. Because each species has a different degree of tolerance to the environment, the suitable habitat threshold for each species was divided based on the maximum value of the available distribution records. The growth suitability at each sampling point was extracted from the plot of the calculated growth suitability. The standard deviation σ and mean value μ were calculated according to the theory of normal distribution; μ‐σ was selected as the threshold value for transforming the species distribution probability maps into 0/1 binary distribution maps. The model accuracy was evaluated using receiver operating characteristic (ROC) curves. The area enclosed by the ROC curve and horizontal axis is the AUC value (Hanley & McNeil, 1982), which can be used to measure the strengths and weaknesses of the model. For species with predicted AUC values that are <0.8 or differ significantly from the distribution range of the species in available information, using the ENMeveal package in R creates a series of candidate models by varying parameters (Muscarella et al., 2014). The parameter setting with the smallest Akaike's information criterion (AICc) value was selected as the optimal parameter to calculate the species distribution model, and the model was run again.

The distribution ranges for the 27 species with less than six recorded distribution points defaults to the grid cells where the distribution points were located. The distribution range layer was converted into a 0/1 binary distribution map. Finally, the binary distribution map of 237 species was superimposed on the grid map, and the number of species appearing in a single grid cell was counted to obtain the species richness distribution map.

### 2.3 Analysis of influencing factors of the species richness pattern

#### 2.3.1 Environment variables

Based on previous studies, we selected 12 environmental predictors in four categories to evaluate the factors explaining the distribution pattern of glires species richness in China. The environment variables are as follows:

1. Hydrothermal characteristics: the availability of energy and water can be measured using many indicators, such as temperature, precipitation, and solar radiation (Pandey et al., 2020). We selected annual mean temperature (AMT), annual precipitation (APT), potential evapotranspiration (PET), and actual evapotranspiration (AET) as substitute variables. We extracted AMT and APT from the WorldClim (https://worldclim.org/) database as measures of temperature and water effectiveness variables, PET and AET were obtained from the Global Land Evaporation Amsterdam Model (GLEAM) (https://www.gleam.eu/).
2. Habitat heterogeneity: the mean elevation (MELV), elevation range (ELR), and the number of vegetation types (VEG) within a single grid cell, the most commonly used predictors to represent information on habitat heterogeneity, were selected as habitat heterogeneity factors. These values were obtained from the RESDC.
3. Climate seasonality: temperature seasonality (TES), annual temperature range (ATR), and precipitation seasonality (PRS) were used as proxies for short‐term climate seasonality. All factors were obtained from the WorldClim database.
4. Human factors: we used the human impact index (HII) and human footprint index (HFI) as proxy variables representing human‐induced effects. The HII and HFI data were downloaded from the archives of the Wildlife Conservation Society (http://sedac.ciesin.colum bia.edu/data/).

#### 2.3.2 Data analysis

Prior to regression model construction, variance inflation factors (VIF) and Spearman correlation coefficients were used to detect collinearity between predictors. Collinearity was found between AMT and PET, AMT and MELV, APT and AET, TES and ATR, and HII and HFI (VIF > 5) (Dormann et al., 2013). To minimize the influence of collinearity, PET, MELV, APT, ATR, and HII were removed based on the correlation between the factors and the response variables, so that the VIF values of all variables were <5 and the correlation coefficients between the variables were <0.8.

Regression tree analysis was used as a predictive model for the three response variables (i.e., species richness of all, nonendemic, and endemic) based on the environmental data set described above, longitude, and latitude. This decision‐based approach uses a recursive partitioning algorithm that divides the dependent variable into smaller subsets based on a yes–no response to predictive criterion for each of the independent variables separately; some variables may be used multiple times in the final model, while others may not be used at all (de la Sancha et al., 2020; Grimshaw & Higgins, 2017). The root node is the explanatory variable that accounts for most of the variation found in the response variable. The branches from the root node will continue to split at child nodes (other strong explanatory variables) until a stopping criterion is reached. In our analysis, we used the default values in the rpart function (Therneau & Atkinson, 2022).

Species richness data usually show non‐normal distribution; therefore, our species richness data were log transformed to fulfill normality assumptions before multiple linear regression analysis. To make the model coefficients comparable, the selected environmental factors were standardized (z‐score, standard deviation = 1, mean = 0) in the multiple linear regression. Multiple linear regression used ordinary least squares (OLS) to determine the most appropriate predictors that explain the richness of the three response variables. The backward stepwise selection method was followed to identify the optimal model. The optimal linear regression model was determined using the stepAIC function in R in combination with the Akaike information criterion (AIC) (Appendix S3). Because spatial autocorrelation affects the explanatory power of regression models, the spatial autocorrelation of the residuals of multiple regression models was assessed using Moran's I method. The residuals of the multiple regression models all had significant spatial autocorrelation (*p* < .001); therefore, the spatial linear simultaneous autoregressive error model (SLM) was further developed using the predictor variables from the optimal model. The explanatory power of the predictor variables for species richness was measured using Pseudo‐*R* ^*2*^ (the square of the correlation coefficient between the predicted and actual values of the model for the nonspatial component) (Kissling & Carl, 2007). The relative importance between the predictor variables was also compared using standard regression coefficients.

To evaluate the relative importance of the predictive variable sets, we separated the environmental factors into four distinct predictor sets based on our main research objectives: (a) hydrothermal characteristics (HC), (b) climatic seasonality (CS), (c) habitat heterogeneity (HH), and (d) human factors (HF). Because all predictors were highly correlated, we eliminated collinearity by performing a principal component analysis (PCA) in each prediction set. The squared term of the predictor variable was included in the principal component analysis, considering the nonlinear relationship between the response variable and environmental factors. We extracted the first two principal components of each prediction set as alternative factors for each type of factor, which accounted for 94% of hydrothermal characteristics, 87% of habitat heterogeneity, 98% of climatic seasonality, and 99% of human factors (Table 1). Then, we performed variance partitioning to assess the pure effects of the predictor variables and their joint contributions to better explain the distribution patterns of species richness. Wayne diagrams were used to show various factor sets' pure and shared effects.

**TABLE 1:** Eigenvalues and contribution rate of the environmental factor sets.

Class	Component	Eigenvalue	Contribution rate of variance/%	Cumulative contribution rate of variance/%
Hydrothermal characteristics	PC1	6.90	0.53	0.53
	PC2	0.62	0.41	0.94
Habitat heterogeneity	PC1	2.90	0.45	0.45
	PC2	2.31	0.42	0.87
Climatic seasonality	PC1	4.15	0.64	0.64
	PC2	1.77	0.34	0.98
Human factors	PC1	3.91	0.75	0.75
	PC2	0.09	0.24	0.99

Statistical analysis for this study was performed in R (http://www. r‐project.org). The “psych” R package was used for principal component analysis (Revelle, 2022), the “MASS” R package for optimal model selection (Venables & Ripley, 2002), the “vegan” R package for variance partitioning (Oksanen et al., 2020), and the “spdep” R package for spatial autoregressive model building (Bivand, 2022).

## 3 RESULTS

### 3.1 Species richness patterns of all glires species

**FIGURE 2:**
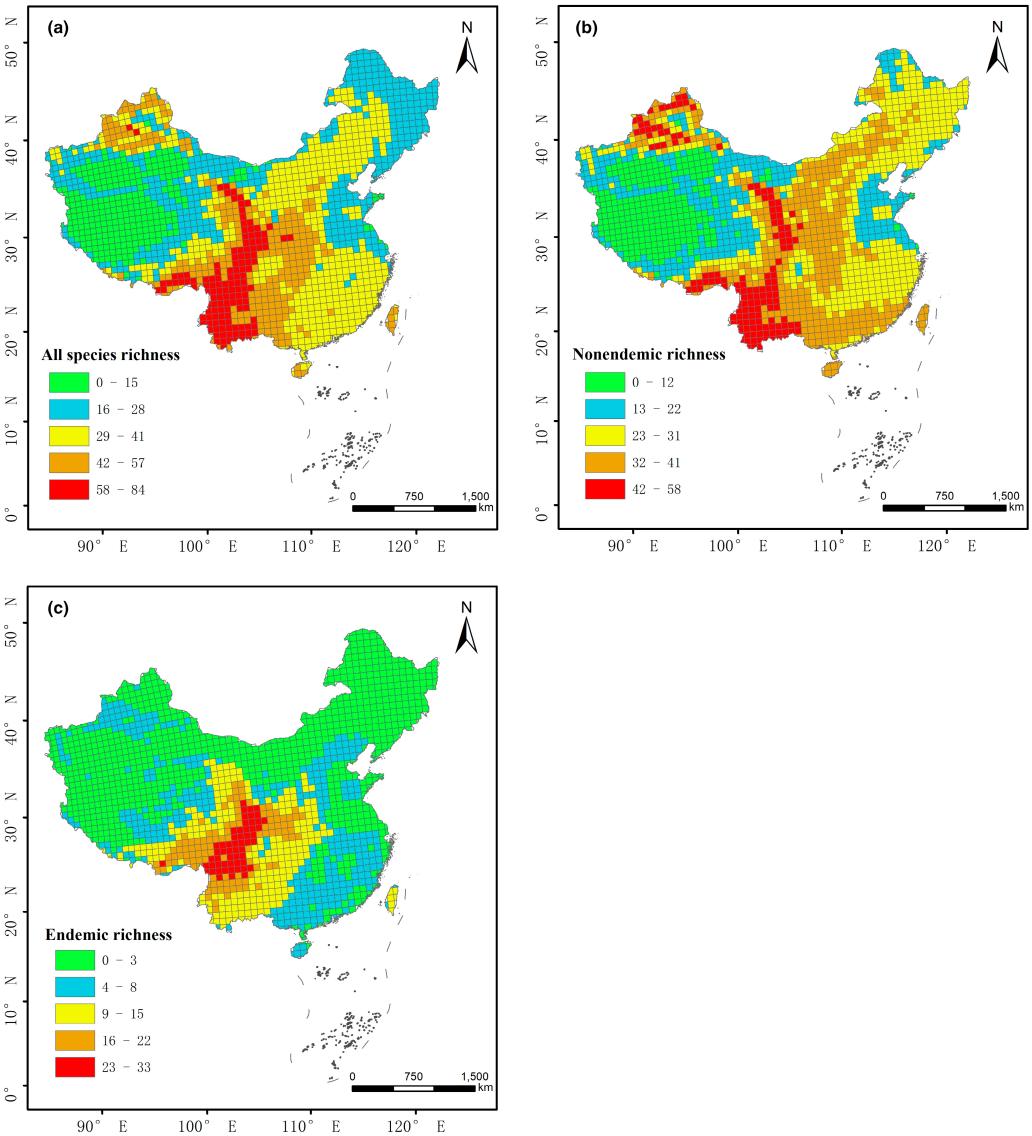
Spatial distribution of glires species in China across all (a), nonendemic (b), and endemic (c) species.

Our results revealed a wide but uneven distribution of glires in China; with at least one species of glires identified in all 1665 grid cells. The species richness in each grid cell was between 0 and 84 (mean: 31.39 ± 16.51 SD) species (Figure 2a; Table 2). We found the highest abundance of glires species in the subtropical and tropical regions of the oriental realm, with the Hengduan Mountains being the most abundant region, followed by the Qilian Mountains and Tianshan Mountains regions. In addition, Taiwan and Hainan Island also had high species richness. The species richness was low in the Qinghai‐Tibet Plateau and Tarim Basin regions, with only a few species in most grid cells.

**TABLE 2:** Descriptive statistics of species richness.

Variables	Total grid	Mean richness per grid	Minimum	Maximum	SD	*n*
All species	1665	31.39	0	84	16.51	237
Nonendemic	1659	25.65	0	58	12.06	170
Endemic	1430	5.75	0	33	6.54	67

**FIGURE 3:**
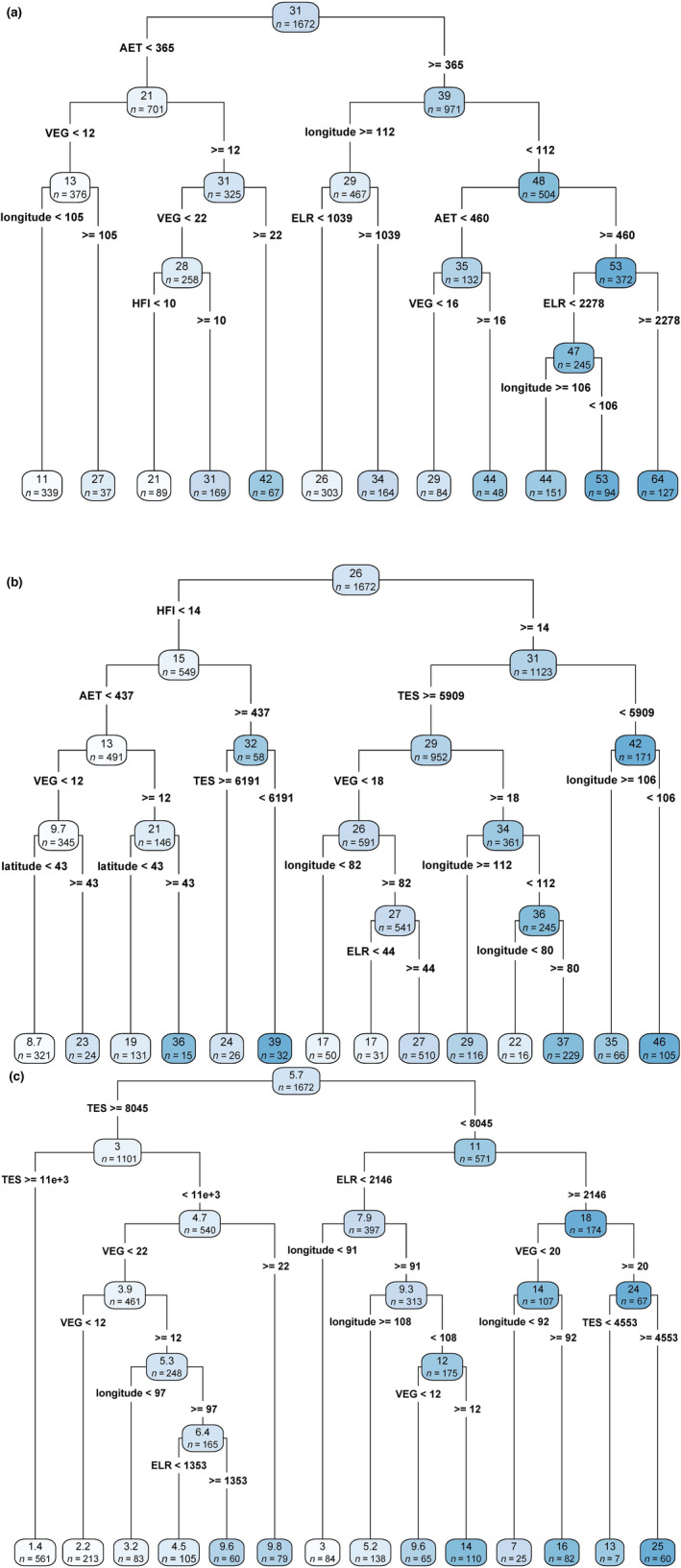
Regression tree of results for species richness for all (a), nonendemic (b), and endemic(c) species of glires. Numbers at terminal tips represent the mean value for grid cells included in that branch; “n” represents the number of grid cells included in that branch. AMT, annual mean temperature; AET, actual evapotranspiration; ELR, elevation range; HFI, human footprint index; PRS, precipitation seasonality; TES, temperature seasonality; VEG, the number of vegetation types.

Our results from regression tree analyses (Figure 3) indicated that the three response variables have different drivers. Actual evapotranspiration (AET) was the main root for all species richness and included in two of the three regression trees. Human footprint index (HFI) was also included in two of the three regression trees and was the root node for nonendemic species richness. Temperature seasonality was found in two of the three trees and was the first node for endemic species richness and the second node for nonendemic species richness. The number of vegetation types (VEG), elevation range (ELR), and longitude were found in all three regression trees but were never the root node. Latitude was only found in the regression tree for nonendemic species richness.

**FIGURE 4:**
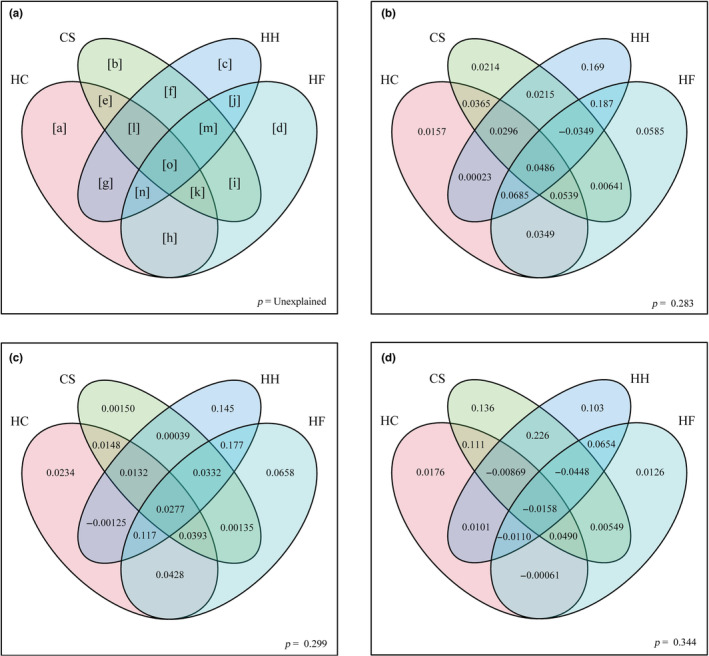
Results of variance partitioning explained by environmental variables (CS, climatic seasonality; HC, hydrothermal characteristics; HF, human factors; HH, habitat heterogeneity). (a) Schematic diagram of variance partitioning, each letter in the figure represents a part of the variance partitioning analysis. Results of variance partitioning for (b) all, (c) nonendemic, and (d) endemic species.

Regarding the relationship between the predictor factors and all species richness, the best model was explained by a set of five variables (AET + TES + ELR + VEG + HFI). The multiple regression model (OLS) explained 66% of the total variation in all species richness. With the removal of the spatial autocorrelation effect by the spatial autoregressive model (SLM), the degree of explanation was reduced to 58% (Table 3). The standardized regression coefficient of the model showed that the importance of each variable in explaining the species richness distribution pattern varied slightly across the regression models. The two models indicated that AET and VEG were the most important predictors, respectively (Table 3). The results of the variation partitioning revealed that the species richness variation explained by all four predictors set was 71.7% for all species. The habitat heterogeneity predictor set explained 48.94% of the variation in all species richness patterns, followed by human factors (42.30%), hydrothermal characteristics (28.79%), and climatic seasonality (18.30%) (Figure 4a,b; Appendix S4).

**TABLE 3:** Results of multiple linear regression (OLS) and spatial autoregressive model (SLM) analysis of glires species richness.

Variable	All species		Nonendemic		Endemic	
	coef_OLS_	coef_SLM_	coef_OLS_	coef_SLM_	coef_OLS_	coef_SLM_
AMT	–	–	0.011	0.003	0.018***	0.004
AET	0.108***	0.010**	0.119***	0.010**	–	–
TES	0.019***	0.004	0.066***	0.011**	−0.240*	−0.021***
PRS	–	–	–	–	–	–
ELR	0.069**	0.025***	0.069***	0.025***	0.084***	0.027***
VEG	0.107***	0.037***	0.096***	0.034***	0.109***	0.033***
HFI	0.065***	0.016***	0.067***	0.018***	0.018*	−0.001
AIC	−1254.6	−3281.7	−1257.5	−3155.7	−36.4	−2353.8
*R* ^2^	0.66	0.58	0.65	0.57	0.66	0.54
Moran's *I*	0.68***	0.072***	0.68***	0.07***	0.74***	0.02

Abbreviations: AET, actual evapotranspiration; AMT, annual mean temperature; ELR, elevation range; HFI, human footprint index; PRS, precipitation seasonality; TES, temperature seasonality; VEG, the number of vegetation types. Moran's *I* is the Moran's index of the regression model residuals. “–” means that the variable is excluded from the optimal model.

* *p* < .05.

** *p* < .01.

*** *p* < .001.

### 3.2 Patterns of nonendemic species richness

Nonendemic species (*n* = 170) accounted for 71.7% of all species and were found in 1659 grid cells. Their distribution in each grid cell ranged from 0 to 58 (mean: 25.65 ± 12.06 SD) species (Figure 2b; Table 2). The species richness distribution pattern of nonendemic species was similar to that of all glires species. The southern Hengduan Mountains, Qilian Mountains, Tianshan Mountains, and Altai Mountains had high nonendemic species richness. Similar to all species, species richness was low in the Qinghai‐Tibet Plateau and Tarim Basin regions.

The best model for predicting nonendemic species richness consisted of six variables (AMT + AET + TES + ELR + VEG + HFI). OLS and SLM explained 65% and 57% of the nonendemic species richness, respectively (Table 3). The difference between the results of the two models was distinct, with a significant difference in the ranking of factor importance. However, both OLS and SLM showed that AMT was the least important predictor. In variance partitioning, all four predictor sets explained 70.10% of the variance in defining the richness patterns of nonendemic species. Nonendemic species richness was significantly correlated with the habitat heterogeneity predictor set, explaining 51.21% of the variation. This was followed by human factors (50.42%), the hydrothermal characteristics predictor set (27.67%), and the climate seasonality predictor set (13.14%) (Figure 4a,c; Appendix S4).

### 3.3 Patterns of endemic species richness

Endemic species (*n* = 67) were found in 1430 grid cells, approximately 28.27% of all species. Endemic species richness in each grid cell ranged from 0 to 33 (mean: 5.75 ± 6.54 SD) species (Figure 2c; Table 2). The distribution of endemic species was predominantly concentrated in the Hengduan Mountains and the surrounding areas. However, their northern distribution is rare, and only a few endemic species adapted to high‐latitude environments.

Regarding the relationship between the distribution pattern of endemic species richness and predictors, the best prediction model contained five variables (AMT + TES + ELR + VEG + HFI). OLS and SLM explained 66% and 54% of the total variation, respectively. Both analyses showed that TES, VEG, and ELR were important influencing factors, but AMT and HFI were relatively less important (Table 3). In variance partitioning, 65.58% of the variance was explained by the four predictor sets. The largest variation in endemic species richness was the total effect of the climatic seasonal predictor set, which explained 45.89% of the variation. The habitat heterogeneity and hydrothermal characteristics predictor sets explained 32.44% and 15.14% of the variation in the endemic species richness pattern, respectively. The human factor predictor set explained the lowest variance (6.03%) (Figure 4a,d; Appendix S4).

## 4 DISCUSSION

Species richness, including that of glires, is characterized by spatial heterogeneity (Gaston, 2000). Our results indicated a wide variety of glires, including endemic species, in the Hengduan Mountains and surrounding areas of southwest China. The Hengduan Mountain area is considered an ecological corridor between the Palaearctic and Oriental realms, connecting the northern and southern fauna. The southwest China is rich in plant resources, has a complex topography and a high degree of habitat heterogeneity. The area's climate ranges from tropical to temperate and is dominated by high temperatures and abundant precipitation (Liu et al., 2015; Shrestha et al., 2017). It is also an important center of diversity (Wu et al., 2016). This may explain why southwest China has the highest level of endemism. In comparison, the extreme climatic conditions (e.g., extremely low temperatures and arid desert) of the Qinghai‐Tibet Plateau, Tarim Basin, and northern China may limit the distribution of species intolerant to climatic factors (Ding et al., 2019; Hu et al., 2017). The results showed that the boundaries of China's Yunnan, Sichuan, and Tibet autonomous regions, especially the Qionglai, Yunling, Minshan, and Gaoligong mountain systems in the Hengduan Mountains, have the highest richness values of all studied areas due to their diverse topography, hydrothermal conditions, and habitats. The distribution pattern of glires species richness may be explained by a combination of habitat heterogeneity, climate seasonality, hydrothermal characteristics, and human factors (Figure 4).

The close relationship between species richness and habitat heterogeneity and the variance partitioning results showed that habitat heterogeneity is the most influential predictor variable for describing the species richness of all and nonendemic glires species in China. It is also an important factor in the richness distribution pattern of endemic species. Habitat heterogeneity is considered the most critical factor in shaping biological distribution patterns. Habitat variation creates a microhabitat for species reproduction by creating dramatic changes in climate and habitat differentiation on a small scale, enabling species to coexist locally (Carmignotto et al., 2022). Southwest China has a variety of hydrothermal conditions and habitats that result from a combination of topographic changes caused by elevation differentiation (Huang et al., 2011; Liu et al., 2015). Consequently, we found that this region had the highest richness values for glires species. In this study, we used the elevation range as one of the main predictors of habitat heterogeneity, arguably the best indicator of topographic variation. Meanwhile, southwest China is also the richest region in terms of plant species (Dakhil et al., 2019; Pandey et al., 2020; Shrestha et al., 2017; Sun et al., 2020). Abundant plant resources provide a large amount of food for glires and a suitable shelter for unfavorable climatic conditions (Barreto et al., 2019). In addition, the Qinghai‐Tibet Plateau's uplift has profoundly changed the geomorphology of mainland China, mountains, ravines, and canyons, resulting in the huge differences on mountain height. Quaternary ice sheet intrusion drives glires migration from higher to lower elevations, and the cyclical effects of global warming cause glires to return to higher elevations (Barreto et al., 2019). Species that cannot migrate can only adapt to the environment of low‐ and medium‐altitude, thus causing high endemic species richness in glires. In addition, highly heterogeneous tropical and subtropical mountain ranges may be cradles of biodiversity and thus dominate in terms of species richness and concentrations of endemic species with narrow distributions.

The results showed that climatic seasonality is the dominant factor in the distribution patterns of endemic species richness of glires in China. Conversely, it explained the least of all the species and nonendemic species richness patterns. Previous research has also identified a significant role of climate seasonality in maintaining patterns of species richness in endemic small mammal species (Amori et al., 2011; Hu et al., 2017; Wu et al., 2013). The temperature in China fluctuates widely, with more extreme temperatures in the north than in the south. This might be an important factor in northern China's restricted distribution of endemic species. Our findings support the theory of tropical niche conservation and the inability of endemic glires species to adapt to northern environments (Berriozabal‐Islas et al., 2018). The ability of glires to migrate from south to north is consistent with our findings (Romdal et al., 2013). This seasonality in southwest China creates stable climatic conditions for endemic species to survive under harsh conditions. Araújo et al. (2008) and Dakhil et al. (2019) reported the significant role of climate stability during the warmest season of the Quaternary glaciation. Climatic stability is an ecological indicator of the range stability of subtropical fauna in the high‐altitude regions of southwest China, including the eastern Tibetan Plateau (Huang et al., 2011; Thuiller, 2004). Because there is much climate variability and unsystematic variation in daily temperatures differences, this modifies the thermal environment experienced by nonendemic glires. As evolutionary response they may have developed an increased physiological tolerance to these temperatures variations, enabling them to be widely distributed geographically (Ramírez‐Bautista et al., 2020). Glires are closely associated with habitat and microhabitat attributes, and they are generally considered to be least affected by the direct effects (e.g., physiological stresses) of climate change (Buckley et al., 2012). This may be the reason why nonendemic species can be widely distributed.

Our results also revealed the importance of anthropogenic variables in the distribution patterns of all and nonendemic species richness. However, their effect on endemic species richness was minimal. Thus, it can be expected that anthropogenic activities will significantly impact the distribution of glires. Anthropogenic activities can drive the contraction and expansion of species. Many species have lost significant areas of distribution owing to increased land use and other human activities (Ceballos & Ehrlich, 2002; Di Marco & Santini, 2015). Human activities have also contributed to the range expansion of several other species, and many regional native species have experienced increased range through human‐mediated dispersal and the ability to thrive in anthropogenic landscapes (Li et al., 2015). However, endemic species have more specialized habitat requirements, are concentrated in specific geographic areas, and may deviate ecologically from a wide range of species (Tomašových & Jablonski, 2016). Therefore, human activities have not yet had much impact on the endemic species. However, endemic species are confined to limited areas, and their habitats are often fragile and highly susceptible to reduction or even disappearance by external factors. Therefore, we cannot overlook human‐interference scenarios. Anthropogenic disturbances such as natural resource exploitation, tourism, and land use change are gradually increasing in southwest China, accelerating the impact of human activities on wildlife. Therefore, protecting this center of species richness and endemism is a big challenge.

Our findings showed that hydrothermal characteristics did not have a significant direct relationship with the distribution pattern of glires species richness in China on a large spatial scale. However, this differs slightly from the results of Hu et al. (2017) and Wu et al. (2013) on distribution at the smaller, local scale. The spatial pattern of species richness and the importance of biotic and abiotic predictor variables in influencing species richness may depend to substantial extent on the spatial scale of the sampling unit (Qian & Kissling, 2010). In some cases, species richness patterns and potential drivers can be reasonably explained by ecological requirements and the evolutionary history of species grouping (Wu et al., 2013). Glires are phytophagous or omnivorous; therefore, the pattern of species richness may be closely related to plant species richness (Hawkins & Pausas, 2004). At a large spatial scale, the direct impact of hydrothermal characteristics on plants is often more significant (Zhang et al., 2015). The effect of hydrothermal characteristics on the distribution pattern of glires species richness is not particularly significant at large scales; however, on a smaller scale, glires dispersal behavior is temperature‐oriented, and species may spread more rapidly when temperature changes (Wu et al., 2013). Therefore, temperature and precipitation may strongly influence glires species richness at a regional scale.

In summary, our findings, consistent with those of previous studies, advise against using all species or nonendemic species richness as proxies for endemic species richness (Isik, 2011; Orme et al., 2005). The reasons for the formation of endemic and widespread species differ. Endemic species formation is often closely related to environmental and anthropogenic changes. Some endemic species may have been widely distributed; however, owing to climate change during the geological and historical period, their habitats have shrunk or even disappeared. Thus, their distribution has been confined to a narrow range. Alternatively, newly evolved species have not yet spread to a larger geographic area, or destruction of the environment limits their ranges. In addition, we found that the reasons for determining the distribution of nonendemic and endemic species richness differed.

Although this study contributes to a more nuanced understanding of glires species richness patterns and their drivers in China, it has several limitations. For example, there are 271 species of glires in China (Wei et al., 2021); however, only the 237 species with relatively complete data were analyzed in this study, which did not provide a complete picture of the glires distribution pattern in China. The variables used in MaxEnt partially overlapped with the factors involved in the regression model, which inevitably affected the regression results. In addition, the species distribution model assumed that species distribution was influenced only by environmental variables, without interactions between organisms and biological dispersal constraints. In practice, species distribution is also influenced by biological factors such as competition, predation, and disease, which can lead to errors in the predicted range of species.

## 5 CONCLUSION

This study investigated the spatial patterns of glires species richness in China and possible interpretations of the results based on multiple environmental and human factors. This study found that the highest species richness values of nonendemic and endemic species of glires in China were in and around the Hengduan Mountains in southwest China. The lowest number of endemic species was in the Qinghai‐Tibet Plateau and northern China. Habitat heterogeneity was the most effective variable to reflect changes in the potential distribution of all glires and nonendemic species, as well as species richness in China. Climatic seasonality was the best predictor set to determine the richness distribution pattern of endemic species, whereas this was least affected by human factors. Furthermore, it should be noted that hydrothermal characteristics were not strong predictors of richness patterns for all or nonendemic species, which may be due to the fact that there are also more species in some areas with less precipitation or energy. Therefore, glires are likely to persist in areas with characteristics of high habitat heterogeneity and stable climate.

## AUTHOR CONTRIBUTIONS

**Lei Meng** was involved in data curation (lead), formal analysis (equal), methodology (equal), visualization (equal), writing—original draft (lead), Writing—review & editing (lead). **Lizhi Zhou** was involved in conceptualization (lead), resources (lead), supervision (lead), visualization (equal), writing—review & editing (equal).

## FUNDING INFORMATION

This work was supported by the National Science & Technology Fundamental Resources Investigation Program of China (Grant No. 2019FY101804).

## CONFLICT OF INTEREST

All authors state that there is no conflict of interest.

## Supporting information


Appendix S1‐S4

## Data Availability

All the predictor variables were extracted from online database and are cited in the manuscript. Data with species richness information are archived in Dryad Digital Repository: https://doi.org/10.5061/dryad.2jm63xstb.
