# Supplementary material for: Distribution patterns and drivers of nonendemic and endemic glires species in China
Source: Ecol Evol. 2023 Feb 7;13(2):e9798. doi: 10.1002/ece3.9798 (PMC9905661; doi:10.1002/ece3.9798)
Supplement: Supplementary file 1 — Appendix S1‐S4 [file ECE3-13-e9798-s001.docx]

**Appendix 1**

Appendix 1 Environment variables used for species distribution model of glires

| Variable type | Environmental variables | Definition | Source |
| --- | --- | --- | --- |
| Climate variables | bio1 | Annual Mean Temperature | WorldClim2.1 （https://worldclim.org/） |
|  | bio2 | Mean Diurnal Range |  |
|  | bio3 | Isothermality |  |
|  | bio4 | Temperature Seasonality |  |
|  | bio5 | Max Temperature of Warmest Month |  |
|  | bio6 | Min Temperature of Coldest Month |  |
|  | bio7 | Temperature Annual Range |  |
|  | bio8 | Mean Temperature of Wettest Quarter |  |
|  | bio9 | Mean Temperature of Driest Quarter |  |
|  | bio10 | Mean Temperature of Warmest Quarter |  |
|  | bio11 | Mean Temperature of Coldest Quarter |  |
|  | bio12 | Annual Precipitation |  |
|  | bio13 | Precipitation of Wettest Month |  |
|  | bio14 | Precipitation of Driest Month |  |
|  | bio15 | Precipitation Seasonality |  |
|  | bio16 | Precipitation of Wettest Quarter |  |
|  | bio17 | Precipitation of Driest Quarter |  |
|  | bio18 | Precipitation of Warmest Quarter |  |
|  | bio19 | Precipitation of Coldest Quarter |  |
| Soil variable | ST | Soil type | Resource and Environment Science andData Center （https://www.resdc.cn/） |
| Topographic variable | ELEV | Elevation above sea level |  |
|  | SLOP | Slope |  |
|  | APSE | Aspect |  |
| Vegetation variable | VT | Vegetation types |  |
| Human variable | HII | Human Influence Index | Socioeconomic Data and Applications Center (sedac) （http://sedac.ciesin.colum bia.edu/data/） |
|  | HFI | Human Footprint Index |  |

**Appendix 2**

Appendix 2 Spearman coefficient of the retained environment variable

|  | bio3 | bio7 | bio12 | bio13 | bio17 | APSE | ELEV | SLOP | ST | VT |
| --- | --- | --- | --- | --- | --- | --- | --- | --- | --- | --- |
| bio3 | 1 | <0.001 | 0.067 | <0.001 | <0.001 | <0.001 | <0.001 | <0.001 | <0.001 | <0.001 |
| bio7 | -0.1093 | 1 | <0.001 | <0.05 | <0.001 | <0.001 | 0.069 | <0.01 | <0.001 | <0.05 |
| bio12 | -0.1391 | 0.1143 | 1 | 0.054 | <0.001 | 0.104 | <0.01 | <0.001 | <0.001 | <0.001 |
| bio13 | -0.0172 | -0.1518 | 0.1318 | 1 | <0.05 | <0.001 | <0.05 | <0.05 | 0.216 | <0.001 |
| bio17 | -0.4394 | 0.3291 | 0.3426 | 0.0557 | 1 | <0.01 | <0.001 | <0.001 | <0.001 | <0.001 |
| APSE | -0.1948 | -0.4569 | -0.4624 | -0.0023 | -0.4363 | 1 | <0.001 | <0.01 | <0.01 | 0.110 |
| ELEV | 0.6010 | 0.2922 | 0.1401 | -0.0521 | -0.1795 | -0.6592 | 1 | <0.001 | <0.001 | 0.053 |
| SLOP | 0.0032 | 0.0347 | 0.0034 | -0.0066 | 0.0053 | -0.0137 | 0.0270 | 1 | <0.001 | <0.05 |
| ST | -0.6543 | 0.4099 | 0.2097 | -0.0448 | 0.7317 | -0.1303 | -0.4472 | 0.0023 | 1 | <0.001 |
| VT | 0.3348 | 0.3529 | 0.0249 | -0.1245 | -0.1434 | -0.4468 | 0.6815 | 0.0137 | -0.2781 | 1 |

Note：The upper right corner is the *P* value

**Appendix 3**

Appendix 3 Results of model selection relating species richness to environmental factors using Akaike Information Criterion (AIC)

| **Species richness** | **Model** | **AIC** | **Adjusted R^2^** |
| --- | --- | --- | --- |
| All species | AMT + AET + TES + PRS + ELR + VEG + HFI | -1247.2 | 0.66 |
|  | AET + TES + PRS + ELR + VEG + HFI | -1250.8 | 0.66 |
|  | AET + TES + ELR + VEG + HFI | -1254.6 | 0.66 |
| Non-endemic | AMT + AET + TES + PRS + ELR + VEG + HFI | -1251.6 | 0.65 |
|  | AMT + AET + TES + ELR + VEG + HFI | -1257.5 | 0.65 |
| Endemic | AMT + AET + TES + PRS + ELR + VEG + HFI | -30.2 | 0.66 |
|  | AMT + AET + TES + ELR + VEG + HFI | -32.7 | 0.66 |
|  | AMT + TES + ELR + VEG + HFI | -36.4 | 0.66 |

AMT = annual mean temperature; AET = actual evapotranspiration; PRS = precipitation seasonality; TES = temperature seasonality; ELR = elevation range; VEG = the number of vegetation types; HFI = human footprint index

**Appendix 4**

Appendix 4 Results of variation partitioning showing the percentage contributions of predictor variables to determine the species richness of Glires. The lowercase letter denotes the individual contribution (adjusted *R^2^*) following the labels displayed in Figure 4a (main text).

| Individual  contribution (%) | Species richness | | |
| --- | --- | --- | --- |
|  | **All species** | **Non-endemic** | **Endemic** |
| [a] | 1.567 | 2.335 | 1.761 |
| [b] | 2.142 | 0.15 | 13.636 |
| [c] | 16.868 | 14.495 | 10.274 |
| [d] | 5.846 | 6.579 | 1.263 |
| [e] | 3.652 | 1.475 | 11.088 |
| [f] | 2.15 | 0.039 | 22.647 |
| [g] | 0.023 | -0.125 | 1.006 |
| [h] | 3.49 | 4.289 | -0.061 |
| [i] | 0.641 | 0.135 | 0.549 |
| [j] | 18.717 | 17.709 | 6.542 |
| [k] | 5.385 | 3.929 | 4.901 |
| [l] | 2.962 | 1.318 | -0.869 |
| [m] | -3.49 | 3.322 | -4.478 |
| [n] | 6.852 | 11.681 | -1.101 |
| [o] | 4.856 | 2.771 | -1.581 |
| [p] = residuals | 28.338 | 29.899 | 34.424 |
| Total hydrothermal characteristics set [aeghklno] | 28.788 | 27.672 | 15.144 |
| Total climatic seasonality set [befiklmo] | 18.299 | 13.138 | 45.893 |
| Total habitat heterogeneity set [cfgjlmno] | 48.94 | 51.211 | 32.44 |
| Total human factors set [dhijkmno] | 42.298 | 50.415 | 6.034 |
